# Proteomics dataset: The colon mucosa from inflammatory bowel disease patients, gastrointestinal asymptomic rheumatoid arthritis patients, and controls

**DOI:** 10.1016/j.dib.2017.09.059

**Published:** 2017-10-06

**Authors:** Tue Bjerg Bennike, Thomas Gelsing Carlsen, Torkell Ellingsen, Ole Kristian Bonderup, Henning Glerup, Martin Bøgsted, Gunna Christiansen, Svend Birkelund, Vibeke Andersen, Allan Stensballe

**Affiliations:** a Department of Health Science and Technology, Aalborg University, Aalborg, Denmark; b AH Diagnostics, Aarhus, Denmark; c Department of Rheumatology, Odense University Hospital, Odense, Denmark; d Diagnostic Center, Section of Gastroenterology, Regional Hospital Silkeborg, Silkeborg, Denmark; e University Research Clinic for Innovative Patient Pathways, Aarhus University, Aarhus, Denmark; f Department of Clinical Medicine, Aalborg University, Aalborg, Denmark; g Department of Haematology, Aalborg University Hospital, Aalborg, Denmark; h Department of Biomedicine, Aarhus University, Aarhus, Denmark; i Institute of Regional Health Research-Center Soenderjylland, University of Southern Denmark, Odense, Denmark; j Department of Internal Medicine, Regional Hospital Viborg, Viborg, Denmark

**Keywords:** Colon mucosa, Proteomics, Rheumatoid arthritis, Ulcerative colitis, Inflammatory bowel diseases, Neutrophil extracellular traps, Dataset, Sigmoidoscopy, Colonoscopy

## Abstract

The datasets presented in this article are related to the research articles entitled “Neutrophil Extracellular Traps in Ulcerative Colitis: A Proteome Analysis of Intestinal Biopsies” (Bennike et al., 2015 [1]), and “Proteome Analysis of Rheumatoid Arthritis Gut Mucosa” (Bennike et al., 2017 [2]). The colon mucosa represents the main interacting surface of the gut microbiota and the immune system. Studies have found an altered composition of the gut microbiota in rheumatoid arthritis patients (Zhang et al., 2015; Vaahtovuo et al., 2008; Hazenberg et al., 1992) [5], [6], [7] and inflammatory bowel disease patients (Morgan et al., 2012; Abraham and Medzhitov, 2011; Bennike, 2014) [8], [9], [10]. Therefore, we characterized the proteome of colon mucosa biopsies from 10 inflammatory bowel disease ulcerative colitis (UC) patients, 11 gastrointestinal healthy rheumatoid arthritis (RA) patients, and 10 controls. We conducted the sample preparation and liquid chromatography mass spectrometry (LC-MS/MS) analysis of all samples in one batch, enabling label-free comparison between all biopsies. The datasets are made publicly available to enable critical or extended analyses. The proteomics data and search results, have been deposited to the ProteomeXchange Consortium via the PRIDE partner repository with the dataset identifiers PXD001608 for ulcerative colitis and control samples, and PXD003082 for rheumatoid arthritis samples.

## Graphical abstract

**Figure:**
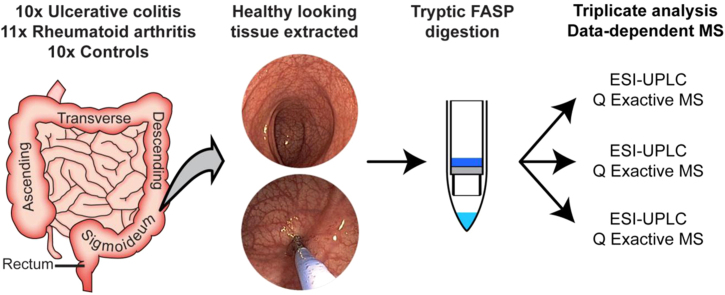

**Specifications Table**

**Table:** 

Subject area	*Biology*
More specific subject area	*Characterization of the proteome of the colon mucosa of ulcerative colitis patients, gastrointestinal healthy rheumatoid arthritis patients, and controls.*
Type of data	*Raw- mass spectrometry files and text/excel files*
How data was acquired	*Mass Spectrometry Liquid Chromatography*
	*Data was acquired using a high-resolution/high-accuracy Q Exactive plus (Thermo Scientific) mass spectrometer*.
Data format	*Raw- and analyzed data.*
Experimental factors	*Human colon mucosal biopsies from ulcerative colitis patients, gastrointestinal healthy rheumatoid arthritis patients, and controls.*
Experimental features	*Biopsies were extracted by colonoscopy and immediately snap-frozen with liquid nitrogen. The biopsies were tryptic digested and analyzed by electrospray ionization liquid chromatography mass spectrometry.*
Data source location	*The Laboratory for Medical Mass Spectrometry, Department of Health Science and Technology, Aalborg University, Fredrik Bajers Vej 7E, 9220 Aalborg East, Denmark*
Data accessibility	The proteomics data have been deposited to the ProteomeXchange Consortium via the PRIDE partner repository, [13], [14], [15], [16] with dataset identifiers:
	PXD001608 – Ulcerative colitis patients, and controls.
	PXD003082 – Gastrointestinal healthy rheumatoid arthritis patients.
	Direct download links: http://www.ebi.ac.uk/pride/archive/projects/PXD001608
	http://www.ebi.ac.uk/pride/archive/projects/PXD003082

**Value of the data**

• The dataset contains the largest number of identified human proteins from colon mucosa biopsies as of 2017.
• The dataset was obtained in one batch, allowing for label-free comparison of the colon mucosa of ulcerative colitis patients, rheumatoid arthritis patients, and controls.
• The first dataset of the colon mucosa of gastrointestinal healthy RA patients.
• The datasets can be analyzed for novel proteome effects of disease and treatments.
• The datasets allow for extended statistical analysis, and we encourage such collaborations.

## 1 Data

The datasets in this article provides information on the proteome of the colon mucosa of inflammatory bowel disease patients with ulcerative colitis [1], gastrointestinal healthy rheumatoid arthritis patients [2], and controls. The study was motivated by the finding of an altered composition of the gut microbiota in rheumatoid arthritis patients [5], [6], [7] and inflammatory bowel disease patients [8], [9], [10]. All biopsies were handled on-site by the project group to limit technical variance. The biopsies were randomized, digested using a modified filter-aided sample preparation protein digestion protocol, and analyzed in technical triplicates by high-throughput proteomics on a Q Exactive mass spectrometer. All experimental factors were kept constant, allowing for a label-free quantitative analysis between all samples. The unprocessed proteomics data files (Table 1) and processed search result files (Table 2), have been deposited to the ProteomeXchange Consortium via the PRIDE partner repository, with the dataset identifier PXD001608 for ulcerative colitis and control samples, and PXD003082 for rheumatoid arthritis samples [13], [14].

**Table 1:** Raw-datafiles in PXD001608 and PXD003082. All samples were analyzed in technical triplicates, and all raw-files are named accordingly (e.g. Ctrl_10_3 is the third repeat of control 10). “Poor R” signifies a Pearson's correlation coefficient *R*<0.95 between the technical repeats, and additional data validation is recommended for studies including these datafiles. The number of identified proteins in each replicate is given, wo/w the MaxQuant *match between runs* feature which transfer MS/MS information between different LC-MS/MS analysis. RA: Rheumatoid arthritis, UC: ulcerative colitis, NA: Not available.

**Filename**	**Sample**	**#Proteins matching OFF**	**#Proteins matching ON**	**Dataset ID**	**Comment**
Ctrl_1	Control	4362, 4279, 4241	5967, 5903, 5897	PXD001608	
Ctrl_2	Control	3613, 3607, 3595	5657, 5664, 5723	PXD001608	
Ctrl_3	Control	4203, 4203, 4188	5936, 5952, 5939	PXD001608	
Ctrl_4	Control	4245, 4268, 4191	5863, 5865, 5849	PXD001608	
Ctrl_5	Control	3961, 3881, 3903	5694, 5683, 5632	PXD001608	
Ctrl_6	Control	4290, 4281, 4242	5966, 5959, 5932	PXD001608	
Ctrl_7	Control	4080, 4099, 4097	5856, 5822, 5817	PXD001608	
Ctrl_8	Control	4269, 4325, 4336	5968, 5986, 5966	PXD001608	
Ctrl_9	Control	4560, 4549, 3974	6103, 6126, 6103	PXD001608	
Ctrl_10	Control	3974, 3993, 3972	5781, 5817, 5779	PXD001608	
UC_1	UC	4290, 4229, 4363	5870, 5852, 5907	PXD001608	
UC_2	UC	3328, 3285, 3328	5148, 5101, 5139	PXD001608	
UC_3	UC	4455, 4472, 4482	6068, 6082, 6041	PXD001608	
UC_4	UC	4236, 4005, NA	5685, 5773, NA	PXD001608	UC_4_3 poor R
UC_5	UC	3097, 3174, NA	5051, 5017, NA	PXD001608	UC_5_3 poor R
UC_6	UC	4458, 4424, 4482	6118, 6108, 6115	PXD001608	
UC_7	UC	3657, 3686, 3693	5647, 5626, 5575	PXD001608	
UC_8	UC	3356, 3288, 3303	5237, 5207, 5164	PXD001608	
UC_9	UC	4681, 4700, 4703	6220, 6233, 6223	PXD001608	
UC_10	UC	3762, 3688, 3674	5587, 5557, 5562	PXD001608	
RA_1	RA	3900, 3789, 3755	5658, 5644, 5632	PXD003082	
RA_2	RA	4347, 4141, 4285	6023, 5997, 5973	PXD003082	
RA_3	RA	4654, 4678, 4629	6204, 6179, 6180	PXD003082	
RA_4	RA	4298, 4287, 4267	5933, 5925, 5899	PXD003082	
RA_5	RA	3472, 3521, 3491	5402, 5390, 5344	PXD003082	
RA_6	RA	3545, 3485, 3526	5735, 5742, 5728	PXD003082	
RA_7	RA	4538, 4476, 4459	6057, 6062, 6071	PXD003082	
RA_8	RA	3619, 3439 NA	5317, 5310, NA	PXD003082	RA_8_3 poor R
RA_9	RA	3361, NA, 3427,	5256, NA, 5187	PXD003082	RA_9_2 poor R
RA_10	RA	4417, 4354, 4361	5991, 6010, 6023	PXD003082	
RA_11	RA	3094, 3055, 3051	5005, 5050, 5006	PXD003082

**Table 2:** Additional submitted Search and FASTA files in PXD001608 and PXD003082. RA: Rheumatoid arthritis, UC: ulcerative colitis.

**Filename**	**Dataset ID**	**Content**	**Description**
CombinedTxtFiles.zip	PXD001608	Zipped MaxQuant combined txt folder.	Result of the label-free quantitative analysis of UC and controls in MaxQuant. The content of each file is described in “tables.pdf”.
131008_Swissprot_Human_Ref_ proteome.fasta	PXD001608	Protein FASTA database file.	Protein database used for the UC and controls analysis.
MaxQuantOutput.zip	PXD003082	Zipped MaxQuant combined txt folder.	Result of the label-free quantitative analysis of UC, RA, and controls in MaxQuant. The content of each file is described in “tables.pdf”.
UniprotHumanProteome P000005640Isoforms.fasta	PXD003082	Protein FASTA database file.	Protein database used for the UC, RA, and controls analysis.
FASTA file parameters.txt	PXD003082	FASTA info.	Information regarding the database.

A cumulated 6768 proteins (FDR<1%) were identified, representing the largest proteome dataset of the colon mucosa so far. Additionally, the dataset represents the first analysis of the colon mucosa of gastrointestinal healthy rheumatoid arthritis patients. The data-analysis result from the analysis with MaxQuant can be downloaded as zipped txt-files, the context of which are described in the tables.pdf also in the zipped file. The result-file proteinGroups.txt, contains all identified proteins at <1% FDR, and information regarding each protein, e.g. the corresponding label-free relative quantitation value (LFQ). Additional information regarding the participants can be found in the publications.

## 2 Experimental design, materials and methods

### 2.1 Study cohort and sample collection

The sample material was extracted and processed as described in [1] and 2].

Colon mucosal biopsies (roughly 1 mm^3^) were sampled 40 cm from the anus by sigmoidoscopy, at the Regional Hospital Silkeborg Denmark, from 10 ulcerative colitis patients, 11 rheumatoid arthritis patients and 10 controls in the period from 2012 to 2013. The biopsies were immediately transferred to cryotubes and snap-frozen in liquid nitrogen followed by storage at minus 80 °C until proteomics sample preparation. All participants had given a written informed consent prior to participation in the study, and the project was approved by The Regional Scientific Ethical Committee (S-20120204) and the Danish Data Protection Agency (2008-58-035).

### 2.2 Proteomic sample preparation

The biopsies were randomized, and enzymatic digested using a modified filter-aided sample preparation protein [17], [18], [19], [20], [21], [22]. Briefly explained, the biopsies were homogenized in 0.5 mL cold sample buffer (5% sodium deoxycholate, 50 mM triethylammonium bicarbonate, pH 8.5). The lysate protein concentration was estimated by absorbance at 280 nm measured using a NanoDrop 1000 UV–vis spectrophotometer (Thermo Scientific, Waltham, MA, USA). Additionally, the concentration of four biopsy lysates was determined using a bicinchoninic acid assay (BCA) with bovine serum albumin as standard, measured using an Infinite microplate reader (Tecan, Männedorf, Switzerland). The nanodrop measurements were calibrated using the BCA results. 100 µg protein was transferred to 30 kDa molecular weight cutoff spin-filters (Millipore, Billerica, MA, USA) to facilitate buffer exchanges by centrifugation at 15,000*g* for 15 min between all steps. Protein disulfide bonds were reduced by addition of 100 µL 10 mM tris(2-carboxyethyl)phosphine (Thermo Scientific, Waltham, MA, USA) and alkylated by addition of 100 µL 50 mM 2-iodoacetamide (Sigma-Aldrich, St. Louis, MO, USA) in sample buffer. Two µg sequencing grade modified trypsin (Promega, Madison, WI, USA) diluted in lysis buffer with 0.5% sodium deoxycholate was added to the spin-filter, and the proteins were digested to peptides overnight at 37 °C. The peptide material was eluted from the spin-filter and purified by phase inversions with 1:1 (v/v) ethyl acetate with 1% formic acid, and dried down in a vacuum centrifuge overnight, and stored at −80 °C for a maximum of one week prior to analysis.

### 2.3 Proteomic analysis

The peptides were analyzed by LC-MS/MS using an UltiMate 3000 UPLC system (Thermo Scientific, Waltham, MA, USA) coupled online to a Q Exactive plus mass spectrometer (Thermo Scientific). Five µg peptide material was loaded onto a 2 cm reverse phase C18-material trapping column and separated on a 50 cm analytical column, both from Acclaim PepMap100 (Thermo Scientific). The liquid phase consisted of 96% solvent A (0.1% formic acid) and 4% solvent B (0.1% formic acid in acetonitrile), at a flow rate of 300 nL/min. The peptides were eluted from the column by increasing to 8% solvent B and subsequently to 30% solvent B on a 225 min ramp gradient, and introduced into the mass spectrometer by a picotip emitter for electrospray ionization (New objective, Woburn, MA, USA). The mass spectrometer was operated in positive mode with data-dependent acquisition, alternating between survey spectra and isolation/fragmentation spectra using a top12 method. Selected eluting peptides were excluded from re-analysis for 30 s. All biopsies were analyzed in triplicates in a random order.

### 2.4 Data processing

The generated RAW-files were searched with MaxQuant 1.5.2.8 software against the Uniprot Homo sapiens reference proteome database with isoforms (UP000005640, last modified 2015-01-16, entry count 90,434) [23], [24]. Standard settings were employed, with the following abundant peptide modifications included in the search: Carbamidomethylated(C) (fixed), N-terminal protein acetylation (variable), oxidation(M) (variable), and deamidation (N or Q) (variable) [11], [12]. The *match between runs* feature in MaxQuant was enabled to allow the transfer of confident peptides identifications across LC-MS/MS runs, based on accurate mass-to-charge and retention time. Identified proteins and peptides were filtered to <1% false discovery rate [25]. Label-free quantitation was enabled in MaxQuant to report protein and peptide relative quantities using standard parameters.

## Funding sources

The Lundbeck Foundation Denmark (R181-2014-3372), and the Carlsberg Foundation (CF14-0561) are acknowledged for grants enabling the project (TBB grants). Knud and Edith Eriksens Memorial Foundation (“Knudog Edith EriksensMindefond”) and Ferring are acknowledge for grants, enabling the collection of the biological sample material (VA grant). The Obelske Family Foundation and the Svend Andersen Foundation are acknowledged for grants supporting the analytical platform being part of the Danish National Platform for Proteomics (PRO-MS) (AS grants).

## References

[1] Bennike T.B. (2015). Neutrophil extracellular traps in ulcerative colitis: a proteome analysis of intestinal biopsies. Inflamm. Bowel Dis..

[2] Bennike T.B. (2017). Proteome analysis of rheumatoid arthritis gut mucosa. J. Proteome Res..

[5] Zhang X. (2015). The oral and gut microbiomes are perturbed in rheumatoid arthritis and partly normalized after treatment. Nat. Med..

[6] Vaahtovuo J., Munukka E., Korkeamäki M., Luukkainen R., Toivanen P. (2008). Fecal microbiota in early rheumatoid arthritis. J. Rheumatol..

[7] Hazenberg M.P., Klasen I.S., Kool J., Ruseler-van Embden J.G., Severijnen A.J. (1992). Are intestinal bacteria involved in the etiology of rheumatoid arthritis? Review article. Acta Pathol. Microbiol. Immunol. Scand..

[8] Morgan X.C. (2012). Dysfunction of the intestinal microbiome in inflammatory bowel disease and treatment. Genome Biol..

[9] Abraham C., Medzhitov R. (2011). Interactions between the host innate immune system and microbes in inflammatory bowel disease. Gastroenterology.

[10] Bennike T. (2014). Biomarkers in inflammatory bowel diseases: current status and proteomics identification strategies. World J. Gastroenterol..

[11] Bennike T.B. (2016). Comparing the proteome of snap frozen, RNAlater preserved, and formalin-fixed paraffin-embedded human tissue samples. EuPA Open Proteom..

[12] Bennike T.B. (2016). Proteome stability analysis of snap frozen, RNAlater preserved, and formalin-fixed paraffin-embedded human colon mucosal biopsies. Data Brief.

[13] Vizcaíno J.A. (2014). ProteomeXchange provides globally coordinated proteomics data submission and dissemination. Nat. Biotechnol..

[14] Vizcaíno J.A. (2013). The PRoteomics IDEntifications PRIDE database and associated tools: status in 2013. Nucleic Acids Res..

[15] Wang R. (2012). PRIDE Inspector: a tool to visualize and validate MS proteomics data. Nat. Biotechnol..

[16] Côté R.G. (2012). The PRoteomics IDEntification PRIDE Converter 2 framework: an improved suite of tools to facilitate data submission to the PRIDE database and the ProteomeXchange consortium. Mol. Cell. Proteom..

[17] Leon I.R., Schwammle V., Jensen O.N., Sprenger R.R. (2013). Quantitative assessment of in-solution digestion efficiency identifies optimal protocols for unbiased protein analysis. Mol. Cell. Proteom..

[18] Wisniewski J.R., Zougman A., Nagaraj N., Mann M. (2009). Universal sample preparation method for proteome analysis. Nat. Methods.

[19] Masuda T., Tomita M., Ishihama Y. (2008). Phase transfer surfactant-aided trypsin digestion for membrane proteome analysis. J. Proteome Res..

[20] Manza L.L., Stamer S.L., Ham A.-J.L., Codreanu S.G., Liebler D.C. (2005). Sample preparation and digestion for proteomic analyses using spin filters. Proteomics.

[21] Bennike T. (2014). A normative study of the synovial fluid proteome from healthy porcine knee joints. J. Proteome Res..

[22] Bennike T., Lauridsen K.B., Olesen M.K., Andersen V., Birkelund S., Stensballe A. (2013). Optimizing the identification of citrullinated peptides by mass spectrometry: utilizing the inability of trypsin to cleave after citrullinated amino acids. J. Proteom. Bioinform..

[23] Cox J., Hein M.Y., Luber C.A., Paron I., Nagaraj N., Mann M. (2014). Accurate proteome-wide label-free quantification by delayed normalization and maximal peptide ratio extraction, termed MaxLFQ. Mol. Cell. Proteom..

[24] Cox J., Mann M. (2008). MaxQuant enables high peptide identification rates, individualized p.p.b.-range mass accuracies and proteome-wide protein quantification. Nat. Biotechnol..

[25] Cox J., Neuhauser N., Michalski A., Scheltema R.A., Olsen J.V., Mann M. (2011). Andromeda: a peptide search engine integrated into the MaxQuant environment. J. Proteome Res..

